# Long-Term Cola Intake Does Not Cause Evident Pathological Alterations in the Femoral Bone Microstructure: An Animal Study in Adult Mice

**DOI:** 10.3390/nu15030583

**Published:** 2023-01-22

**Authors:** Veronika Kovacova, Radoslav Omelka, Vladimira Mondockova, Piotr Londzin, Jozef Conka, Veronika Meliskova, Joanna Folwarczna, Peter Celec, Monika Martiniakova

**Affiliations:** 1 Department of Zoology and Anthropology, Faculty of Natural Sciences and Informatics, Constantine the Philosopher University in Nitra, 949 01 Nitra, Slovakia; 2 Department of Botany and Genetics, Faculty of Natural Sciences and Informatics, Constantine the Philosopher University in Nitra, 949 01 Nitra, Slovakia; 3 Department of Pharmacology, Faculty of Pharmaceutical Sciences in Sosnowiec, Medical University of Silesia, Katowice, 41-200 Sosnowiec, Poland; 4 Institute of Molecular Biomedicine, Faculty of Medicine, Comenius University, 811 08 Bratislava, Slovakia; 5 Institute of Physiology, Faculty of Medicine, Comenius University, 811 08 Bratislava, Slovakia

**Keywords:** cola intake, bone microstructure, bone mechanical properties, bone health, adult mice

## Abstract

Short-term animal experiments and association studies in humans have shown that cola intake may have a detrimental impact on bone mineral density (BMD); however, other bone parameters have not been investigated. This study examined the effects of long-term cola consumption on the femoral bone microstructure using adult mice (*n* = 32) as an animal model, which were divided into water and cola groups depending on whether they received water or cola along with a standard rodent diet for 6 months. Micro-computed tomography revealed that cola intake did not significantly affect all measured parameters characterizing trabecular bone mass and microarchitecture, as well as cortical microarchitecture and geometry in both sexes, although a slight deterioration of these parameters was noted. Cola consumption also resulted in a slightly, statistically insignificant worsening of bone mechanical properties. In contrast to female mice, males receiving cola had a lower area of primary osteons’ vascular canals. Nevertheless, long-term cola intake did not cause evident pathological alterations in the femur of adult mice, possibly due to a balanced diet and no restriction of physical activity. Therefore, the adverse effects of cola consumption on BMD, the only bone parameter studied so far, may be caused by other risk and lifestyle factors.

## 1. Introduction

In recent years, total consumption of sugar-sweetened beverages (SSB) including carbonated soda soft drinks, such as cola, sweetened bottled waters, fruit juices, sports and energy drinks is rising dramatically [1,2,3]. Consumption of these beverages may have a detrimental impact on human health [2]. Numerous epidemiological studies have revealed that intake of SSB is associated with overweight and obesity [4,5], type 2 diabetes mellitus [6], metabolic syndrome [7], bone damage [8,9], dental caries [3,10], and cardiovascular diseases [11,12,13]. With regard to the bone, short-term animal experiments performed on rats have shown that cola intake may have an adverse impact on bone mineral density (BMD) [10,14]. Additionally, association studies in humans have determined a negative relationship between cola consumption and BMD [8,15]. Cola beverages include high amounts of phosphorus (P), caffeine, and fructose corn syrup, reducing bone mineral accumulation, which can further lead to bone loss and increased fracture risk [8,10,14,16]. The effect of cola consumption on BMD may also be sex dependent, creating an asymmetry in this parameter between males and females [15]. Except for BMD, the relationship between other bone parameters and cola intake has not been investigated in experimental animals and/or humans.

In general, bone is characterized by a complex hierarchical structure from macro- to nano-scale collagen and mineral components. The macro-scale is the whole-bone level, whilst the meso-scale is represented by cortical and trabecular structures. At the micro-scale, the cortical bone is composed of secondary osteons with a central Haversian canal and a cement line, whereas trabeculae constitute a porous network in the trabecular bone. Both cortical and trabecular bone are formed by micrometric lamellae, composed of bundles of collagen fibrils. Inside aforementioned fibrils there are lacunae, sub-micrometric cavities, where osteocytes reside. At the nano-scale, collagen fibrils are composed mainly of type I collagen molecules and hydroxyapatite nanocrystals [17,18,19]. While macro- and meso-scale imaging finds application in clinical practice, micro- and nano-scale imaging is primarily used for research purposes to extract fragility indexes [20].

Research on various bone-related diseases has shown that factors other than BMD (assessed using dual-energy X-ray absorptiometry; DXA) also play important roles in maintaining bone health and mechanical integrity. These may include three-dimensional parameters of the trabecular bone, such as bone volume fraction, trabecular number and thickness as well as the orientation and connectivity of trabeculae. Parameters of cortical microarchitecture may incorporate bone volume fraction, cortical bone thickness, BMD as well as those associated with breaking strength and resistance to torsion, e.g., polar moment of inertia or section modulus (Imax/Cmax, Imin/Cmin). Two-dimensional parameters of the cortical bone, such as the area of vascular canals, secondary osteons, population density of secondary osteons may be related to blood supply and bone toughness. The mechanical behavior of bone (e.g., determination of the yield point load, maximum load and fracture load) is a multifaceted subject relevant to studies of fracture, development, adaptation and regeneration. Deterioration of bone mechanical properties can have serious consequences for individuals’ quality of life. Therefore, our study focused on investigating possible changes in the microstructure and mechanical properties of the femur in adult male and female mice after long-term cola intake by analyzing all aforementioned parameters. The use of mice as model organisms to study human biology is based on genetic and physiological similarities between these species. The mouse is a suitable commonly used animal model for human disease research [21]. We hypothesized that long-term cola intake may adversely affect most bone parameters examined in our experiment.

## 2. Materials and Methods

### 2.1. Animals and Experimental Procedures

Healthy adult (3-month-old) CD-1 mice (♂; *n* = 16; ♀; *n* = 16; Velaz, Praha, Czech Republic) were used in this study. Animals were acclimated for 1 week before starting the experiment. Mice were housed 4 per standard polycarbonate cage with a shared floor area of 375 cm^2^. Temperature (20–24 °C), relative humidity (40–60%), and the photoperiod (12-h/12 h light/dark) were monitored and regulated. Mice from the water (W) group (W♂, *n* = 8; W♀, *n* = 8) had ad libitum access to standard rodent commercial diet and tap water, while mice from the cola (C) group (C♂, *n* = 8; C♀, *n* = 8) had free access to the rodent diet and cola (decarbonated Coca cola from local store) for 6 months. The main components of the feed mixture (1240.7 kJ/100 g) included carbohydrates (54.3 g/100 g), proteins (14.1 g/100 g), and fats (1 g/100 g). The diet also contained 1.1% calcium (Ca) and 0.8% P. The local version of Coca cola contained 11.2 g per 100 mL saccharides in the form of a fructose-glucose sirup, 61.4 mg per 100 mL of phosphoric acid, and 9.7 mg per 100 mL of caffeine. The average daily liquid intake was twice as high in the C group (13 mL per mouse) in comparison to the W group (6 mL per mouse). Additionally, reduced food intake was observed in the C group (4 g per mouse and day) versus W group (6 g per mouse and day). Stress from crowding, social isolation, or immobilization was prevented.

### 2.2. Macroscopic Indicators

After 6 months, all mice were sacrificed under complete anesthesia (Ketamine/Xylazine—100 mg/kg and 10 mg/kg body weight) by exsanguination and their body weights (BW), femoral weights (FW) and femoral lengths (FL) were recorded.

### 2.3. Micro-Computed Tomography

Micro-computed tomography (micro-CT, μCT 50, Scanco Medical, Brüttisellen, Switzerland) was used to evaluate cortical and trabecular bone microarchitecture. High resolution scans with a voxel size of 6.8 μm were acquired. The region of interest for cortical bone analysis was selected at a distance of 5.2 mm from the end of the growth plate (EGP) and extending 1.5 mm at the femoral midshaft, whereas that for trabecular bone analysis was selected at a distance of 1.2 mm from the EGP and extending 1.5 mm. Scanning parameters included 70 kVp voltage, 200 mA current, 300 ms integration time, 0.5 mm aluminum filter. Noise reduction in the scans was achieved using a Gaussian filter (sigma = 0.8; support = 1). Microstructural parameters were determined by micro-CT software (Scanco Medical). Measurements of cortical microarchitecture and geometry included bone volume fraction (BV/TV, %), bone surface (Bs, mm^2^), BMD (mg HA/ccm), cortical bone thickness (Ct.Th, mm), bone area (BA, mm^2^), polar moment of inertia (pMOI, mm^4^) and two breaking strength parameters that are Imax/Cmax (mm^3^), and Imin/Cmin (mm^3^). Trabecular bone mass measurements involved bone volume fraction (BV/TV, %), bone surface (Bs, mm^2^), BMD (mg HA/ccm), trabecular number (Tb.N, 1/mm), trabecular thickness (Tb.Th, mm), trabecular separation (Tb.Sp), whereas those of trabecular microarchitecture incorporated structure model index (SMI) and connectivity density (Conn.D, 1/mm^3^). BMDs were calculated using a hydroxyapatite phantom (Scanco Medical).

### 2.4. Histomorphometry

For histological analysis of the cortical bone, femurs were cut at the diaphyseal midshaft. Transverse thin sections (70–80 μm) were prepared according to Martiniakova et al. [22] and were visualized under light microscope with polarization (Leica DM 2000, Germany). The quantitative 2D parameters were assessed using Motic Images Plus 2.0 ML (Motic China Group Co., Ltd., Nanjing, China) and Image J [23]. The areas of primary osteons’ vascular canals (POVC), Haversian canals (HC), intact secondary osteons (SO) as well as secondary osteon population density (number of intact secondary osteons per mm^2^, SOPD) were determined in all views (i.e., cranial, caudal, medial, lateral) of thin sections.

### 2.5. Bone Mechanical Properties

Part of the bone samples was used to study mechanical properties. The three-point bending test was performed with the use an Instron 3342 apparatus (measuring range from 0 N to 500 N; Instron, Norwood, MA, USA), connected to a computer with Bluehill 2 software, version 2.14 (Instron, Norwood, MA, USA). The load was applied to femoral diaphysis, perpendicularly to the long axis of the femur, in the mid-length of the bone. The distance between the supporting points was 8 mm. After preconditioning to obtain steady positioning, the mechanical test was started with the displacement rate of 0.01 mm/s and sampling rate of 100 Hz. Based on the load–displacement curves obtained for each bone, the following extrinsic parameters were determined: the load, displacement, and energy for the yield point (0.05% offset), maximum load point, and fracture point. Moreover, the stress for the yield point, maximum load point, and fracture point (intrinsic parameters) were also assessed. For the stress calculation, the femoral diaphysis was assumed to be a circular beam.

### 2.6. Statistical Analysis

All statistical analyses were carried out using SPSS Statistics 28.0 software (IBM, New York, NY, USA). The data were presented as the mean ± standard deviation (SD). To determine differences in bone parameters measured, two-way analysis of variance (ANOVA) with post hoc (Tukey) test was used. The *p* value lower than 0.05 (*), 0.01 (**), and 0.001 (***) was considered to be statistically significant.

## 3. Results

### 3.1. Macroscopic Indicators

**Figure 1:**
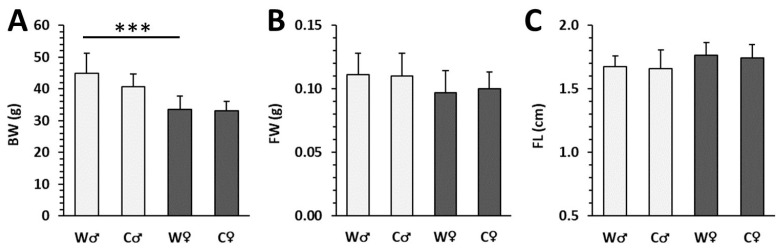
Macroscopic parameters in male (♂) and female (♀) mice from water (W) and cola (C) groups. (**A**) Body weight (BW), (**B**) femoral weight (FW), (**C**) femoral length (FL). The values are represented as mean ± SD. Only differences between groups W♂ and W♀ as well as W and C of the same sex are indicated (*** *p* < 0.001).

Male mice from the water group (W♂ mice) had increased BW compared to females (W♀ mice; Figure 1A). On the other hand, no significant differences in FW and FL were determined between sexes (W♂ and W♀ individuals; Figure 1B,C). Cola intake did not cause significant changes in BW, FW, FL between the W and C (cola) groups of either sex (Figure 1A–C), potentially due to reduced food intake when drinking cola.

### 3.2. Micro-Computed Tomography

**Figure 2:**
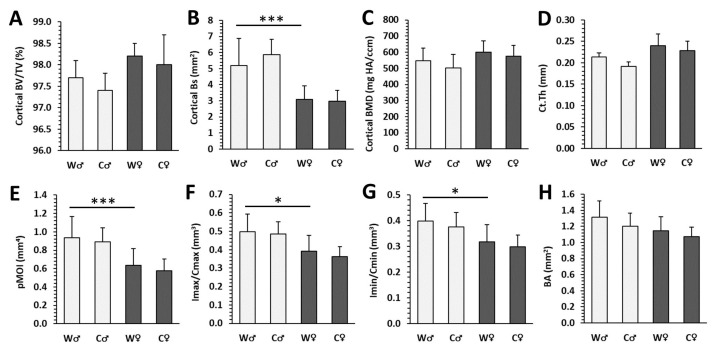
Micro-CT cortical parameters in male (♂) and female (♀) mice from water (W) and cola (C) groups. (**A**) Volume fraction (bone volume/total volume; BV/TV), (**B**) bone surface (Bs), (**C**) bone mineral density (BMD), (**D**) cortical bone thickness (Ct.Th), (**E**) polar moment of inertia (pMOI), (**F**) maximum loading resistance (Imax/Cmax), (**G**) minimum loading resistance (Imin/Cmin), (**H**) bone area (BA). The values are represented as mean ± SD. Only differences between groups W♂ and W♀ as well as W and C of the same sex are indicated (* *p* < 0.05, *** *p* < 0.001).

**Figure 3:**
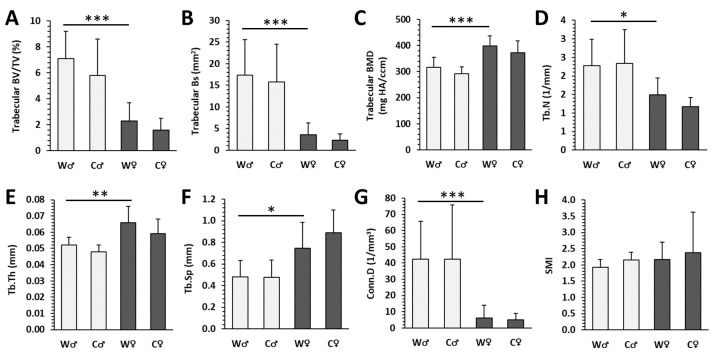
Micro-CT trabecular parameters in male (♂) and female (♀) mice from water (W) and cola (C) groups. (**A**) Volume fraction (bone volume/total volume; BV/TV), (**B**) bone surface (Bs), (**C**) bone mineral density (BMD), (**D**) trabecular number (Tb.N), (**E**) trabecular thickness (Tb.Th), (**F**) trabecular separation (Tb.Sp), (**G**) connectivity density (Conn.D), (**H**) structure model index (SMI). The values are represented as mean ± SD. Only differences between groups W♂ and W♀ as well as W and C of the same sex are indicated (* *p* < 0.05, ** *p* < 0.01, *** *p* < 0.001).

**Figure 4:**
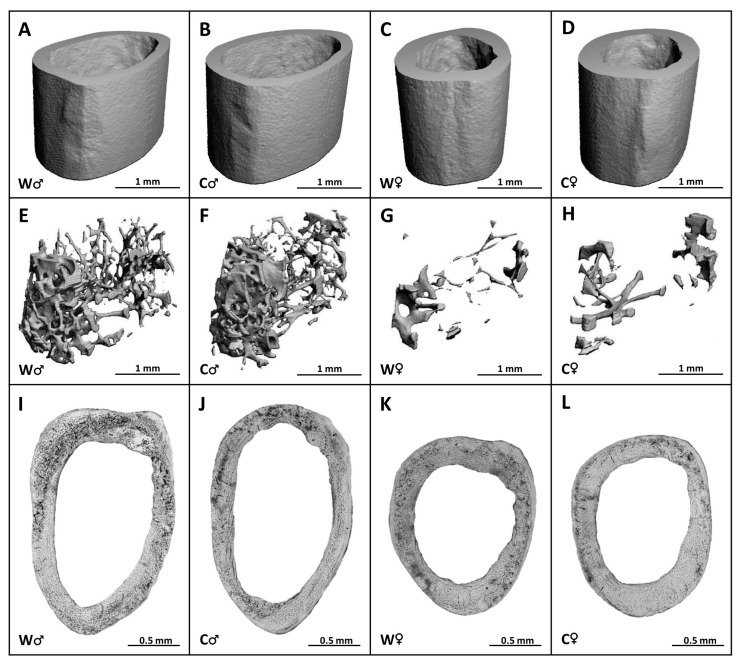
Micro-CT images of the cortical (**A**–**D**) and trabecular (**E**–**H**) bone and microscopic images of the cortical bone (**I**–**L**) in male (♂) and female (♀) mice from water (W) and cola (C) groups.

Male mice from the W group had significantly higher cortical Bs, pMOI, Imax/Cmax, Imin/Cmin (Figure 2B,E–G), as well as trabecular BV/TV, Bs, Tb.N, Conn.D (Figure 3A,B,D,G) versus females (W♀ mice). In female W individuals, significantly elevated trabecular BMD, Tb.Th, and Tb.Sp (Figure 3C,E,F) were recorded versus male W mice. These data indicate sex-related changes in the femoral bone microstructure in the W group. Cola consumption did not significantly affect any of the measured parameters in cortical and trabecular bone compartments of the femur in both sexes, although a slight deterioration of these parameters was noted (Figure 2 and Figure 3). Representative reconstructed 3D images are shown in Figure 4A–H.

### 3.3. Histomorphometry

**Figure 5:**
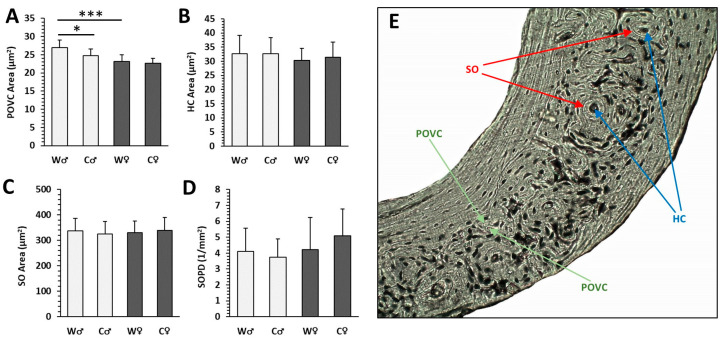
Histomorphometrical parameters in male (♂) and female (♀) mice from water (W) and cola (C) groups. (**A**) Primary osteons’ vascular canals (POVC) area, (**B**) Haversian canals (HC) area, (**C**) secondary osteons (SO) area, (**D**) secondary osteon population density (SOPD). The values are represented as mean ± SD. Only differences between groups W♂ and W♀ as well as W and C of the same sex are indicated (* *p* < 0.05, *** *p* < 0.001). (**E**)—Specific histological image showing all measured parameters (magnification ×200).

**Figure 6:**
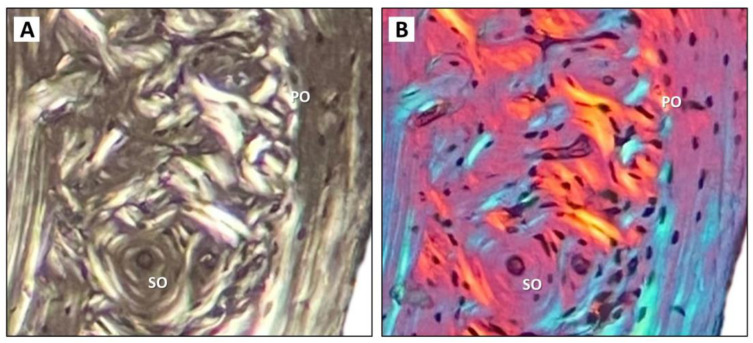
Histological images showing the pattern of collagen fibers determined using polarized light microscopy (**A**) and with the addition of λ plate compensator (**B**). Primary osteon (PO), secondary osteon (SO) (magnification ×400).

Mice from both W and C groups had similar cortical bone microstructure. Periosteal and endosteal surfaces were formed mainly by circumferential lamellae and osteocyte lacunae. Vascular canals, both branching and non-branching, radiating from the marrow cavity were observed near the endosteal border. In the central area of the cortical bone and below the periosteum, several SO with a few concentric lamellae were found. Primary osteons (PO) were more common (Figure 4I–L and Figure 5E). The pattern of collagen fibers determined using polarized light microscopy and a λ plate compensator is shown in Figure 6. The area of POVC was significantly higher in male W mice compared to female W mice (Figure 5A). Interestingly, male mice from the C group had a lower area of POVC (about 8%) versus male W individuals (Figure 5A). On the contrary, areas of HC and SO were not affected by cola intake in both male and female mice (Figure 5B,C). SOPD was not significantly different between the W and C groups of both sexes, and this parameter was not influenced by cola administration as well (Figure 5D).

### 3.4. Bone Mechanical Properties

**Figure 7:**
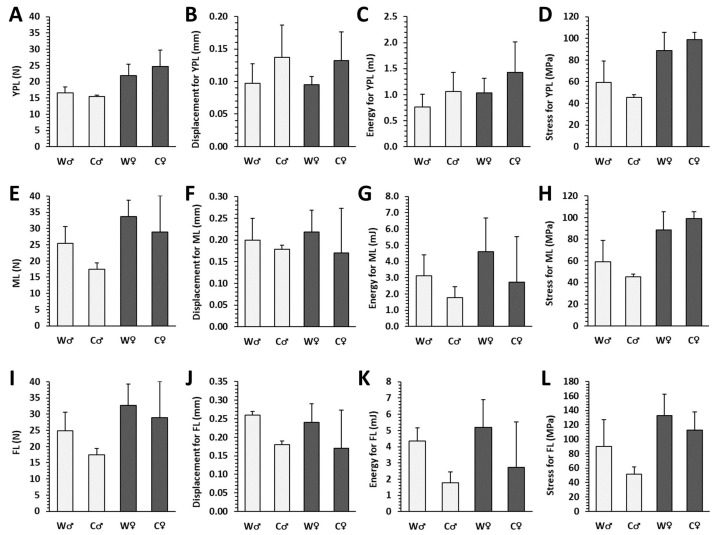
Mechanical properties of left femurs in male (♂) and female (♀) mice from water (W) and cola (C) groups. (**A**–**D**) Yield point load (YPL) displacement, energy and stress, (**E**–**H**) maximum load (ML) displacement, energy and stress, (**I**–**L**) fracture load (FL) displacement, energy and stress. The values are represented as mean ± SD.

No significant changes in all measured parameters were noted between male and female mice from the W group. Cola administration resulted in a slightly, statistically insignificant worsening of bone mechanical properties (Figure 7).

## 4. Discussion

This study demonstrates that long-term cola intake does not cause obvious pathological alterations in the cortical and trabecular bone compartments of the femur and mechanical properties in adult mice. The impact of prolonged cola intake was evaluated only between individuals of the same sex due to evident sex- and age-related changes in murine bone microstructure. In mammals, skeletal sexual dimorphism generally occurs [24]. It is determined not only by gonadotropic axis (androgen action in males and estrogen action in females), but depends on gender related differences in somatotropic axis (growth hormone/insulin-like growth factor 1 action) and mechanical loading as well [25,26]. The aforementioned dimorphism was also confirmed in the W group in our study. Sex-related differences in bone microstructure have also been recorded by Gautam et al. [27] (in Tb.N of mice; values were decreased in females), Glatt et al. [28] (in trabecular BV/TV, Tb.N, Conn.D of mice; values were decreased in females), Martiniakova et al. [29] (in sizes of POVC of rabbits, values were decreased in females), Khosla et al. [30] (in trabecular BV/TV, Tb.Th of humans, values were decreased in young females), and by Kazakia et al. [31] (in Ct.Th of humans, values were decreased in females). On the other hand, some studies do not report any changes in the gender-associated bone parameters listed above, such as in Ct.Th of mice [32], Tb.N and, Ct.Th of humans [30]. These discrepancies may be due to analyses of other skeletal segments, the different species used, and/or other technologies used for measurements. In addition, with increasing age, apparent differences in trabecular bone microarchitecture between the sexes may occur, which was also confirmed in our study between male W and female W mice. In C57BL/6J mice, age-associated decreases in trabecular BV/TV, Tb.N, total body BMD were more pronounced in females than males [28]. The aforementioned changes in mice are similar to those recorded in humans. Young women had lower trabecular BV/TV than men, and with increasing age, women had a greater decrease in BMD, Tb.N, and a smaller reduction in Tb.Th [30,33].

In our experiment, BW, FW, and FL were not affected by cola consumption in both sexes. No significant differences in BW have also been determined in ApoE−/− mice receiving cola for 8 weeks [34]. Similarly, cola intake did not cause changes in BW of adult male rats [35] as well as ovariectomized (OVX) rats [14] after 3 and 2 months, respectively. Contrariwise, in the study by Choi et al. [36], cola administration was associated with weight loss in weaning male rats, receiving simultaneously a moderate fat diet (for 28 weeks) due to reduced insulin resistance with increased skeletal muscle glucose uptake. In humans, BW of healthy adults of both sexes did not differ significantly after ingestion of beverages with sucrose or intense sweeteners during 10 weeks [37].

Short-term animal experiments and epidemiological studies in humans pointed to the association between cola intake and decreased BMD [8,10,14,38,39]. Taking rodents into account, significantly reduced BMD has been determined in adult rats [10] as well as in OVX rats [14] receiving cola for 30 days and 2 months, respectively. These experiments showed that cola intake might induce hypocalcemia and glomerular damage leading to a decrease in BMD. In contrast, our results demonstrated that prolonged cola intake did not affect BMD in adult mice of either sex. An unchanged BMD has also been recorded in adult as well as older women with moderate intake of carbonated beverages [16,40]. These women had modest Ca intake, but not significantly reduced. In contrast, women who consumed cola regularly had significantly lower Ca intake and a lower Ca:P ratio, which may have contributed to reduced BMD. In men with similar Ca intake, no significant effect of cola consumption on BMD was found, but they had higher physical activity than women [8].

These results show that many risk and lifestyle factors may be consistent with lower BMD in humans [41,42]. Generally, individuals drinking more carbonated soft drinks also consume foods with lower nutritional values, reduced Ca content, and higher glycaemic index [43] which together can contribute to BMD variation. Some studies report that increased P intake (also through cola consumption) is detrimental to bone health in people whose dietary Ca:P ratio is extremely low [44,45]. On the contrary, there is a strong evidence that high P administration does not adversely affect Ca balance in individuals with adequate Ca and P supplementation [46,47]. According to Heaney [48], there is no evidence that caffeine (found in cola beverages) has any detrimental effect on bone health or Ca economy in individuals receiving the current recommended daily allowances of Ca. In connection with that, no association between caffeine intake and BMD was reported in healthy premenopausal women [49,50,51]. Similarly, no significant effects of chronic caffeine intake on Ca or P metabolic balances were determined in adult Sprague Dawley rats [52]. Therefore, the impact of caffeine on Ca/P metabolism could be small enough even in our mice receiving cola. The animals used in our experiment received sufficient amount of Ca, their dietary Ca:P ratio was adequate, and all important nutrients were included in their diet. They did not have any physical activity restrictions. Except for BMD, other bone parameters evaluated by micro-CT cannot be compared with published data due to their absence. Anyway, our results suggest that long-term cola intake did not significantly affect trabecular bone mass and microarchitecture as well as cortical microarchitecture and geometry in adult mice of either sex.

Our findings from histological analysis of the cortical bone in both W and C groups are consistent with other researches, which on the one hand indicate the possibility of intracortical bone remodeling, but on the other hand confirm the predominance of parallel lamellae over concentric ones in rodents [22,53,54,55,56]. Several SO we identified lacked numerous concentric lamellae and did not show the highly organized networks observed in humans [54]. Except for SO, more PO were observed in the cortical bone. PO are formed by the incorporation of a periosteal vessels network during appositional bone growth, and their central canal area is determined by the size of the capillary-like vessel [57,58]. PO have only a few lamellae and do not have cement lines, because they are not the product of bone remodeling [59]. Their existence can be related to body size and rapid growth [60]. Significantly decreased area of POVC in male C mice may indicate decreased blood supply in these bone structures. In the bone, immature and mature osteoblast populations interact clearly with bone vascularization [61]. Vascular endothelial growth factor (VEGF) plays a central role in linking angiogenesis and osteogenesis. The study by Goring et al. [62] suggests that bone-derived VEGF regulates matrix mineralization and vascularization clearly in males and females, and systemic alteration of VEGF could affect bone differently in both sexes. Therefore, important ingredients in cola drinks (e.g., glucose, caffeine) could act on the bone via VEGF. It is known that high glucose levels significantly suppress VEGF expression in multipotent rat bone marrow progenitor cells, presumably through inhibition of JAK2/STAT3 (Janus kinase 2/signal transducer and activator of transcription 3) signaling [63]. On the other hand, sex hormones also have a different effect on VEGF production. While estradiol increases VEGF expression by osteoblasts [64], testosterone decreases VEGF production in bone marrow mesenchymal stem cells [65]. Celec and Behuliak [66] revealed that consumption of caffeine-containing cola drinks over 3 months was associated with elevated plasma testosterone and estradiol levels in adult male rats. In epidemiological studies, caffeine intake was consistent with higher estrogen levels and lower free testosterone in postmenopausal women [67]. Moreover, caffeine is able to affect estrogen metabolism in women [68,69]. Based on findings mentioned above, the combination of these factors and their consequences at the physiological level could contribute to different blood supply in POVC of male C mice. No significant differences in the area of SO and their density between the W and C groups of either sex are in agreement with the results from micro-CT (pMOI, Imax/Cmax, Imin/Cmin) as well as those from three-point bending test (e.g., YPL, ML and FL), which together show that prolonged cola intake does not significantly influence cortical bone toughness and strength of adult mice. It is known that a decreased size of SO can be related to an increased risk of fragility fractures [70]. Larger SO are geometrically more advantageous for resisting bending and compressive loads [71]. According to Evans and Vincintelli [72] and Lanyon et al. [73], the presence of SO increases the fatigue life of the cortex, as it can help reduce the severity of microdamages. Cement lines in SO have the ability to reduce the energy of microdamage, thereby limiting their propagation [74].

It should also be mentioned that proper renal function contributes to healthy bones. In our study, significant pathological alterations in the femoral bone microstructure and mechanical properties related to cola consumption were not reported, probably due to well-functioning kidneys. It is known that the kidneys, as a major organ involved in the regulation of mineral homeostasis (e.g., Ca and P), play a key role in regulating bone mineralization, metabolism and development [75,76]. In this context, 3-month cola intake had no impact on renal function and glomerular morphology parameters in adult male rats [35]. In addition, Cao et al. [77] revealed a protective role of physical activity on renal disorder in adult male cola-drinking rats. Therefore, a balanced diet and no restriction of physical activity in our mice can help eliminate the possible harmful effects of cola intake on the kidneys and subsequently on bones.

Our hypothesis has not been confirmed, since long-term cola intake significantly affected only one parameter in the cortical bone microstructure of adult male mice. However, this change was not explicitly pathological. In adult female mice, no significant differences in both femoral bone microstructure and mechanical properties were determined. These results suggest that the harmful impacts of cola administration on BMD, as the only bone parameter studied to date, may be caused by other risk and lifestyle factors in published studies. However, our study has some limitations. First, further experiments focused mainly on molecular and physiological aspects would be appropriate for a more objective explanation of these findings. Second, the development of imaging technologies makes it possible to analyze the 3D geometry of the lacunar-canalicular network at a higher sub-micrometer resolution. Such technologies could thus reveal early signs of eventual bone alterations even in cases where this is not possible using methods with a lower resolution. Focused ion beam/scanning electron microscopy (FIB/SEM), confocal laser scanning microscopy, or synchrotron radiation computed tomography are especially used for these purposes. Due to certain limitations of technologies mentioned above, which include mainly depth range and lower availability, ultra-high-resolution micro-CT technology might be more advantageous [78]. Moreover, recent approaches allow the use of synchrotron scans to quantify bone stress in combination with morphological differences of the lacunar-canalicular network, where bone alterations such as micro-damages can be detected using artificial intelligence [79]. Using finite element models, generated directly from micro-CT or synchrotron images, it is also possible to virtually simulate the mechanical loading of the sample and evaluate the occurrence of bone damage [20].

## 5. Conclusions

Prolonged cola intake did not significantly influence trabecular bone mass and microarchitecture, as well as cortical microarchitecture and geometry in the femur of adult mice, although a slight deterioration of these parameters was noted. Bone mechanical properties were also insignificantly worsened by cola administration. Unlike females, male mice receiving cola had a lower area of the POVC. However, it can be concluded that long-term cola intake did not cause obvious pathological changes in the femoral bone microstructure of adult mice, probably due to a balanced diet and no restriction of physical activity. Further experiments with immature and elderly individuals of both sexes are needed to more objectively assess the impact of cola consumption on bone health.

## Data Availability

The data presented in this study are available on request from the corresponding authors.
